# Welcome to *Medical Gas Research*

**DOI:** 10.1186/2045-9912-1-1

**Published:** 2011-04-27

**Authors:** John H Zhang

**Affiliations:** 1 Editor-in-Chief, Medical Gas Research, Loma Linda University School of Medicine, Loma Linda, California, USA

## Editorial

Medical gas is a large family including oxygen, hydrogen, carbon monoxide, carbon dioxide, nitrogen, xenon, hydrogen sulfide, nitrous oxide, carbon disulfide, argon, helium and other noble gases. These medical gases are used in various disciplines of both clinical medicine and basic science research including anesthesiology, hyperbaric oxygen medicine, diving medicine, internal medicine, emergency medicine, surgery, and many basic science subjects such as physiology, pharmacology, biochemistry, microbiology and neuroscience. Unfortunately, there is not even one journal dedicated to medical gas research at the basic, translational, or clinical sciences level; especially in the neurobiology or neuroscience fields let alone the other various medical fields. Therefore, I am thrilled to introduce this new journal named *Medical Gas Research *to you in this launching editorial.

The aim of *Medical Gas Research *is to publish basic, translational, and clinical studies in biology, especially neurobiology, and their applications with various disorders. Due to the unique nature of medical gas practice, *Medical Gas Research *will also serve as an information platform for education and technology advances for medical gas fields. We hope it will also emphasize novel approaches to help translate the scientific discoveries from basic medical gas research into the development of new strategies for the prevention, assessment, treatment, and enhancement of medical gas clinical applications. *Medical Gas Research *will be an open access journal, in that all articles will be freely and universally accessible online. This open access approach makes an author's work available to readers at no cost and not limited by their library's budget. This ensures that the author's work is disseminated to the widest possible audience than any subscription-based journal either in print or online [1]. Open access may lead to higher downloads and higher citation rates of one's work. In addition, authors hold copyright for their own work and the authors can grant anyone the right to reproduce and disseminate the article. Furthermore, all articles published by *Medical Gas Research *will be archived in PubMed Central and other freely accessible full-text repositories.

Another major strength of *Medical Gas Research *is that to be the first and the leading international journal focusing on medical gas research with eight experienced researchers serving as Associate Editors [2]. This includes Drs. Mark Coburn and Carsten Hobohm who are physician-scientists whose clinical experience with medical gases will enhance the translational aspect of this journal. Professors Shigeo Ohta and Xuejun Sun are world leading researchers in hydrogen and hyperbaric oxygen studies who will play fundamental roles with regards to the basic science aspect of this journal. Dr. Aneesh Singhal, a neurologist at Harvard Medical School, whose basic science, translational, and clinical trial expertise will weigh in tremendously to the quality of studies published. Dr. Atsunori Nakao whose research interests lie beyond the traditional medical field and will bring medical gas research into various directions. Dr. George Mychaskiw, who is currently the Editor-in-Chief of the Undersea and Hyperbaric Association journal and Chairman of Anesthesiology at Drexel University School of Medicine, will serve as the major link between *Medical Gas Research *and other medical gas related journals especially in the field of anesthesiology. And last but not least, Dr. Harry Whelan, a US Navy Officer and Neurology Professor who will bring his own experience with the field to the journal.

Furthermore, another major feature of *Medical Gas Research *is the fifty-member strong editorial board which represents medical gas physicians/researchers from 13 countries around the world [2]. These Editorial Board members will not only provide expertise in clinical, translational, and basic science research in all different medical fields, but many are also practicing anesthesiologists whose clinical knowledge in anesthetics is an important aspect of this journal.

There are two major challenges researchers in the medical gas fields are facing - first is the need for more basic science studies on the fundamental mechanisms of medical gases; and the second is the need for well-planned and executed clinical trials to test the various indications and applications of medical gases [3]. In order to fulfill this aim and the scope of *Medical Gas Research *as mentioned above, the following format will be used for all journal submissions including editorials, commentaries, reviews, clinical and laboratory studies, short communications, case studies, study protocols, meeting reports, medical hypothesis, and letters to the editor. We welcome commentaries and meeting reports that summarize and challenge medical gas research; we welcome review articles that summarize past studies whilst still critiquing the strengths and weaknesses of the study and suggest future research directions; we will publish both clinical case studies and bench experiments either as full articles or as short communications or case reports. In order to keep the medical gas field moving forward, we welcome new methodology, protocol studies and technologies in medical gas applications. And finally as a forum to exchange ideas, *Medical Gas Research *welcomes articles on medical hypothesis and letter to the editors.

We face many exciting challenges down the road, and through this journal we would like to lead the discussion on how to change the form and shape of medical gas into powders or nano-sized bubbles which dissolve well in liquids so that in the future, masks and tubes may not be needed and can be replaced by injections or pills. We also want to lead the discussion on medical gas application devices such as self-propelled or slow releasing implanted "gas tanks" which can be used for chronic conditions and for tissue regeneration. Additionally, through this journal we would like to understand how medical gas promotes tissue protection and re-growth, and to discover specific receptors, signaling pathways, and/or antagonists that can manipulate the local and systemic effects of medical gases. Through all these discussions, we hope to expand medical gas research into agriculture, the airline and space industry, cosmetics, and eventually, make medical gas a normal part of our daily lives.

To send out these messages, we need a stage - *Medical Gas Research *is such a stage that will bring talents from all over the world together to make medical gases useful for the health of human beings.
